# Challenges of data quality for the management of prostate cancer in Tanzania: A hurdle towards improving the quality of service

**DOI:** 10.1371/journal.pgph.0005579

**Published:** 2025-12-04

**Authors:** Obadia Nyongole, Castory Munishi, Njiku Kimu, Raphael Z. Sangeda, Mark Mseti, Emmanuel Lugina, Frank Kiwara, Nathanael Sirili, Frank Chacha, Bartholomeo Nicholaus, Gasto Frumence, Daudi Simba, David Urassa, Bruno Sunguya

**Affiliations:** 1 Department of Surgery, Muhimbili University of Health and Allied Sciences, Dar es Salaam, Tanzania; 2 Department of Community Health, Muhimbili University of Health and Allied Sciences, Dar es Salaam, Tanzania; 3 Department of Pharmaceutics and Pharmacy Practice, Muhimbili University of Health and Allied Sciences, Dar es Salaam, Tanzania; 4 Department of Urology, Muhimbili National Hospital, Dar es Salaam, Tanzania; 5 Department of Pharmaceutical Microbiology, Muhimbili University of Health and Allied Sciences, Dar es Salaam, Tanzania; 6 Ocean Road Cancer Institute, Dar es Salaam, Tanzania; 7 Department of Urology, Mbeya Zonal Referral Hospital, Mbeya, Tanzania; 8 Department of Development Studies, Muhimbili University of Health and Allied Sciences, Dar es Salaam, Tanzania; 9 Department of Urology, Bugando Medical Center, Mwanza, Tanzania; 10 Department of Urology, Kilimanjaro Christian Medical Center, Moshi, Tanzania; WUSM: Washington University in St Louis School of Medicine, UNITED STATES OF AMERICA

## Abstract

Data quality in clinical notes is crucial for providing quality prostate cancer services, especially in countries with weak health systems and a growing burden of non-communicable diseases like prostate cancer. Poor data quality can lead to inefficient services, delayed treatment, and compromised patient outcomes. The objective of this study was to examine the quality of clinical notes for prostate cancer patients treated at five tertiary hospitals in Tanzania from January to December 2022. A sequential mixed-method approach was employed, combining quantitative and qualitative data collection. Patients’ level information from electronic and manual clinical notes were extracted and reviewed. In-depth interviews were conducted among 25 healthcare providers to explore data quality challenges in clinical notes. Quantitative data analysis used descriptive analysis via SPSS 27 software to determine data completeness and accuracy. In-depth interviews were conducted among 25 healthcare providers to explore data quality challenges. Qualitative data analysis utilized thematic analysis with hybrid inductive and deductive reasoning via NVivo 14 software. Clinical notes for prostate cancer management had poor quality. While the overall accuracy of documented variables was high at 1,494 (99.4%), documentation of key clinical variables was low; specifically, the clinical stage was documented in only 1,052 (70.0%) clinical notes, and the Gleason score in 923 (61.4%). Age, clinical presentation, and type of treatment showed high completeness. Themes established included lack of knowledge on data quality, poor integration of data, shortage of human resources for health, lack of supervision and the coexistence of electronic and manual record systems. The Health Information Management System and hospital-based cancer registries were not integrated. Documentation rates for clinical stage and Gleason score in the clinical notes of patients with prostate cancer were low in Tanzania. Improving the quality of clinical notes requires collaborative efforts among stakeholders focusing on structural reforms and capacity building of personnel.

## Introduction

Routine data remains the mainstay for decision-making in health systems of many low- and middle-income countries, LMICs [1,2]. Ensuring data quality is a continuous focus in public health programs, as poor data quality can lead to misleading results, waste of time and resources, and negatively impact treatment outcomes [3–5]. Data quality encompasses several crucial dimensions, including completeness, accuracy, accessibility, relevance, timeliness, and consistency [4,6,7].These dimensions are vital for accurate analysis, providing feedback to enhance operational efficiency, ensuring compliance with regulators, generating customer insights, and supporting forecasting, planning, quality assurance, and governance within the health system [7–9].

In Sub-Saharan Africa (SSA), Health Management Information Systems (HMIS) are often fragmented, which poses significant challenges to data quality, particularly concerning completeness, timeliness, accuracy, and consistency [9,10]. These issues are exacerbated by factors related to human resources, the health system itself, and existing infrastructure [8,11,12]. Despite the introduction of electronic health information systems showing improvements in reporting completeness and timeliness in many countries, Tanzania still lacks an integrated cancer registry and HMIS [1,8,9].

Tanzania, like other low and middle-income countries (LMICs), is experiencing an unprecedented rise in the burden of cancer and other NCDs(6,8). High-quality data in the management and care of patients with these conditions are essential to inform targeted programs and interventions for this epidemiological transition [13,14]. Prostate cancer, being a leading type of cancer among men in Tanzania, particularly relies on good quality data for timely treatment decision-making. Clinical decisions and the course of treatment depend on comprehensive information in clinical notes, including the patient’s clinical type, stage, and grade of disease; demographic information (such as age); clinical presentation; histological results; and the treatment offered throughout the course of care [14–16]. Consequently, the quality of care can be significantly compromised without accurate information from clinical notes to support treatment options and timely decisions [1,6,10,17–20].

Healthcare workers often face challenges such as a poor understanding of the importance of quality clinical notes and their associated variables or indicators, inadequate skills, heavy workloads, and a lack of incentives [8,16]. While a few studies have evaluated the quality of clinical notes in the context of cancer patient services, there is a notable absence of systematic evidence collection to inform policies and guidelines for transforming cancer care in Tanzania [4,21–24]. Therefore, the purpose of this study was to examine the quality of clinical notes for prostate cancer patients treated at five tertiary hospitals in Tanzania from January to December 2022.

### Ethics statement

Ethical clearance was obtained from the Research and Ethics Committee of the Muhimbili University of Health and Allied Sciences (MUHAS) number MUHAS-REC-05-2023-1671. Permission to access and review clinical notes was granted by the respective hospital authority before data extraction. Written informed consent was obtained from each participant before the interview. Patients treated for prostate cancer are required to have signed consent during registration allowing their information in the clinical notes to be used for research without personal identification. Participants’ information was kept confidential even during the analysis and presentation of findings. We used numbers to ensure the anonymity of the source of information according to the General Data Protection Regulation Guideline (GDPR) [25]. To ensure confidentiality, the digital audio recorder was encrypted and protected with a password and stored in a locked cabinet.

## Methods

### Study design

A sequential mixed-method approach was employed, combining quantitative and qualitative data collection [26]. Patient-level information was extracted and reviewed from both electronic and manual clinical notes at five tertiary hospitals of Tanzania from June to November 2023. The quantitative data extraction from clinical notes using a pre-tested checklist was followed by qualitative data collection using in-depth interviews. Qualitative approach was used to explore healthcare providers’ perceptions on the quality of clinical notes of patients treated for prostate cancer in Tanzania. Detailed methods for this study was published in another study [16]. Data triangulation was also used to compare manual clinical notes with electronic electronic clinical notes(data). Duplication of information in clinical notes was assessed specifically in the electronic clinical notes by counting the number of patient clinical notes entered more than once. Data security was assessed by examining the presence of databases for storage of electronic clinical notes storage, the presence and frequency of data backups, and the existence of guidelines for storage of clinical notes and backups. We looked at who had access to the information from clinical notes and the physical security measures in place. While not explicitly quantified as a dimension, the study also explored data consistency between manual and electronic clinical notes despite varying patient numbers across hospitals. We adhered to consolidated criteria for reporting qualitative research similar for mixed methods (COREQ) checklist for improving the overall organization and aligning with reporting standards. S1 Text.

### Study setting

The five tertiary hospitals selected for this study were the referral facilities providing oncology and urology services to patients with prostate cancer in Tanzania. They included three government-owned facilities: Ocean Road Cancer Institute (ORCI), Muhimbili National Hospital (MNH), and Mbeya Zonal Referral Hospital (MZRH); and two faith-based facilities Bugando Medical Centre (BMC) and Kilimanjaro Christian Medical Centre (KCMC). Both ORCI and MNH are in Dar es Salaam, the commercial city in the Eastern zone of Tanzania, while MZRH serves the Southern highlands, located in Mbeya region. BMC serves the lake zone from Mwanza region, while KCMC serves the North-eastern zone from Kilimanjaro region. These hospitals use electronic and manual clinical notes at different levels of care.

### Study population

Study population included healthcare providers involved in the care and handling of patients’ clinical notes. They were purposively selected based on the assigned roles until data saturation was reached. The informants included ten clinicians including urologists and oncologists, nine nurses, and six medical recorders.

### Data sources

The study involved a review of documents, health information systems (HIMS) and hospital-based cancer registry, and clinical notes of patients who were treated for prostate cancer at the selected hospitals. The source documents included clinical notes, pathology reports, hospital registers, tally sheets, and monthly summary reports from the outpatient department (OPD) and the inpatient department (IPD).

### Quantitative data collection

A pre-tested checklist was used to extract information in clinical notes focusing on sociodemographic information, diagnosis, cancer staging, and other data variables. Data collection was conducted using a validated structured checklist in Research Electronic Data Capture (RedCap). The inclusion criteria were presence of a histological report of prostate cancer and evidence of the patients being attended for prostate cancer in 2022.Clinical notes of prostate cancer patients in form of electronic and manual data sources from the medical record department were sorted to exclude duplicates.

### Qualitative data collection

The research team included four experienced health professionals who are faculty at MUHAS with experience in conducting both quantitative and qualitative studies. Moreover, the research team was trained on the study objectives and ethics so that prior assumptions and beliefs could not influence the perception or interpretation of findings. We pretested a semi-structured interview guide after back-to-back translations between English and Kiswahili. In-depth interviews (IDIs) were conducted in Kiswahili and lasted 15–30 minutes. Saturation of information was reached from 23^rd^ to 25^th^ participants. After consenting, a digital audio recorder was used for interviews. A research assistant moderated the interviews, and the principal investigator took field notes at the same time.

### Data management and quality control

Data was collected using a password protected Redcap institutional account, and access was granted to the study personnel only. Quality issues of clinical notes were communicated to the facility personnel to get clarifications and validate whether they were quality issues of clinical notes at the facility rather than data entry errors by the study data collectors. This helped data validation by discerning whether the errors originated at the facility or data entry errors.

### Data analysis

Completeness of information in clinical notes was considered high or acceptable if it reached a 90% level or more. Clinical experts assessed accuracy of information and examined whether the clinical notes were correct for a given variable based on clinical knowledge. Completeness of information in clinical notes was determined by calculating the proportions of complete clinical notes for various key clinical variables compared to the total number of reviewed clinical notes. Quantitative data was analysed on 1,794 outpatients and 806 inpatients’ clinical notes. We examined distribution of variables, missing values, and outliers to ascertain the pattern of the quality of clinical notes. Descriptive analysis for the quantitative component was conducted using Statistical Package for the Social Sciences (SPSS) version 27 software to determine elements of data quality including completeness and accuracy of information in clinical notes.

For qualitative analysis, we adopted the six stages of thematic analysis using hybrid inductive and deductive reasoning approaches through NVivo 14 software pragmatically to accommodate positivist and constructivist views regarding the quality of clinical notes [27]. In-depth interviews (IDIs) were conducted in Swahili and were translated verbatim into English by the first author. Transcribing the audio was followed by coding the interviews for the ownership and drawing insight from the clinical notes. The investigator listened to the audio to detect errors and in some doubtful situations before our participant left the room as part of data cleaning and where necessary, follow-up questions were asked for clarity. Codes were grouped into sub-themes to reflect issues identified by the participants as vital to them for creating sub-themes and themes in executing the analysis work. We used extended involvement, observation and triangulation, comprehensive and detailed explanations, rigorous documentation, and peer debriefing to ensure the trustworthiness of our findings [28,29].

## Results

### Completeness of clinical notes for prostate cancer management at tertiary hospitals in Tanzania

**Fig 1:**
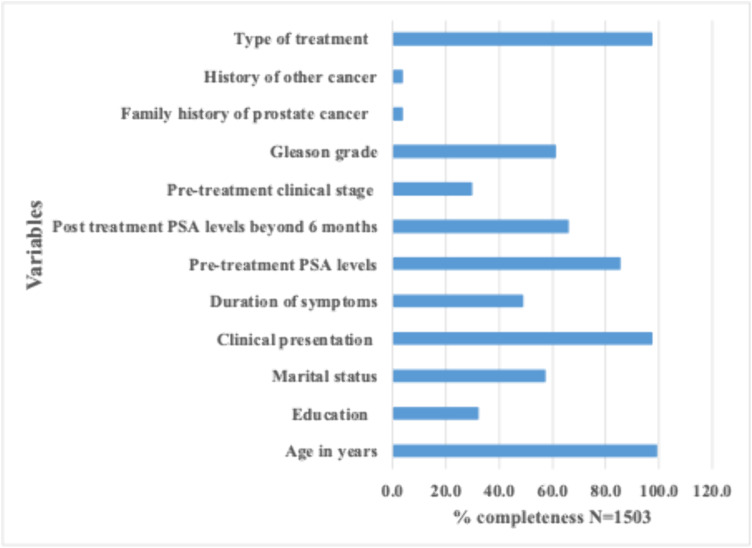
Completeness of clinical notes for prostate cancer management at tertiary hospitals of Tanzania.

Most clinical notes for prostate cancer management had incomplete documentation of key variables. Diagnosis of prostate cancer was documented without a Gleason score in the pathologist report. Completeness of clinical notes was high or acceptable for three of the variables. These were age, clinical presentation, and type of treatment. Documentation of age was excellent in 1493 (99.3%) clinical notes. Education level was documented only in 484 (32.2%) of all reviewed clinical notes. Clinical presentation and type of treatment were documented in 1466 (97.5%) case notes. Pre-treatment PSA was documented in only 1284 (85.4%) clinical notes. Documentation of the clinical stage of prostate cancer was done in only 1052 (70.0%) clinical notes, while the Gleason score was documented in only 923 (61.4%) Fig 1.

A total of 25 healthcare providers were interviewed. Participants were of three cadres which are medical recorders, clinicians, and nurses. Ten of them were also interviewed in the capacity of managers of the hospitals. Their age range was from 38 to 53 years with working experience of more than five years (Table 1).

**Table 1:** Sociodemographic information of qualitative interview participants.

Characteristics	Frequency
**Age (In years)**	
30-40	6 (24%)
41-50	14 (56%)
51-60	5 (20%)
**Education**	
Diploma	7 (28%)
Degree	10 (40%)
Masters	8 (32%)
**Cadre**	
Clinician	10 (40%)
Nurses	9 (36%)
Medical recorder	6 (24%)
**Working experience**	
5 years	2 (08%)
6–10 years	12 (48%)
> 10years	11 (44%)

From qualitative data analysis, we unveiled lack of knowledge regarding quality of clinical notes, poor integration of data causes delays of services delivery, shortage of HRH impairs the quality of clinical notes, lack of proper supervision causes poor quality of clinical notes, coexistence of electronic and manual record systems impairs the quality of clinical notes, the role of proper storage of clinical notes for security, and protection of patients’ clinical notes for quality services (Table 2).

**Table 2:** Summary of qualitative findings.

Codes	Sub-theme	Theme
• Incomplete information in clinical notes • Unreadable sentences in clinical notes • Duplications of clinical notes • Shortage of staff • Lack of supervision	• Poor documentation of variables in clinical notes • Coexistence of electronic and manual record systems impairs the quality of clinical notes • Fragmented HIMS	Contributors to poor quality of clinical notes
• Accuracy of information in clinical notes • Consistency of information in clinical notes • Poor data entry of variables in electronic clinical notes • Data cleaning	• Integrated hospital information management system • Data processing (timeliness) • Data integrity	• Lack of proper supervision causes poor quality of clinical notes
• Unauthorized access to clinical notes • Secure server(backups) • Data protection officer • Consent to the processing of personal data	• Data protection (access) • Storage clinical notes • Security of information • Protection of patients’ information is important for quality services	Proper storage of clinical notes enhances security of information

Medical recorders reported a lack of knowledge on the importance of documentation of key prostate cancer variables. Lack of knowledge regarding quality of clinical notes among healthcare providers, especially medical recorders who may not understand the importance of specific disease variables, contributes to poor documentation.

Demand of training on quality of clinical notes *“I think it is good to have some training on quality of clinical notes to health care providers and colleagues from medical records because medical record officers sometimes do not document variables because he/she does not know the importance of it*.” (HCP-2–06).

Referral letters/reports are required to have comprehensive details about the patient, what was found, what was done, and why the patient was referred. A detailed referral report was reported that it will reduce delays in service provision which is likely to influence positively the treatment outcome. Health information management systems (HIMS) were not integrated between referring hospitals. This lack of integration leads to clinicians having to retake patient histories and repeat investigations done at other referral hospitals, causing unnecessary delays in service delivery. There is a lack of proper supervision and dedicated staff for data cleaning. The coexistence of electronic and manual record systems was reported to impair the quality of clinical notes. This transition phase led to duplication and inconsistencies in data entry.

Lack of platform for integration of clinical notes among hospitals causes delays of services delivery *“We are getting patients who are referred from other tertiary hospitals for radiotherapy with incomplete information, so we start taking history again and this disturbs our patients and delays service delivery*. *It is frustrating to us as clinicians when we start taking the history of a patient who has been attended in another tertiary hospital, I will be happy if we have an integrated system that allows us to access information in the clinical notes about what was done to our patient before referral. This will remove delays in service provision*” (HCP-1–01)

Shortage of human resources for health (HRH), including urologists/oncologists, increases workload and stress, resulting in poor and incomplete documentation, including brief and sometimes unreadable notes in clinical notes. Clinicians reported writing short notes to save time and attend to many patients. Healthcare providers were writing sentences that were not clear but also difficult to read in clinical notes. Follow-up clinics contributed significantly to poor documentation in both manual and electronic clinical notes. Unplanned follow-up clinics with an unrealistic number of patients gave an excuse for proper documentation to the clinician. The shortage of clinicians was reported to contribute to incomplete documentation and even to poorly written sentences because the clinician’s target is to attend to all patients at the clinic for completeness, which is not proper for quality service provision.

Shortage of HRH impairs the quality of clinical notes *“We sometimes write short notes, especially during follow-up clinics, to save time because we have many patients with prostate cancer who are waiting at our clinics, and we are few compared to their number. I am personally forced by the situation to write a summary to save time so that I can attend all prostate cancer sessions at my clinic when I am on duty”* (HCP-2–07).

### Accuracy of information in clinical notes, consistency, and duplication for prostate cancer management at tertiary hospital in Tanzania

Duplication of information in clinical notes was prevalent in the electronic record system, with 1097 clinical notes were entered twice. This was primarily attributed to the transition from manual to electronic clinical notes of which 848(56.4%) were manual clinical notes and 655(44.6%) were electronic clinical notes, where some clinical notes were double-enteredmaking a total of 2600 provided clinical notes.We identified inaccuracies in the clinical notes, such as mislabeling a patient’s sex or diagnosis. For example, some patients with prostate cancer (a male-only disease) were labelled as female. The accuracy score of information in clinical notes were 1494 (99.4%). Inaccuracies were also observed such as mislabeling of the patient’s sex and diagnosis Table 3. This was influenced by a fragmented health information management system (HIMS) and errors that occur during data entry of manual clinical notes to the electronic clinical notes.

**Table 3:** Accuracy of information and duplication in clinical notes.

Hospitals	Given Clinical notes	Meeting criteria	Clinical notes missing	Duplicate clinical notes	Writing error (Sex, diagnosis)	Accuracy (%)
Hospital 1	746	431(57.8%)	0	315(42.2%)	4	99.1%
Hospital 2	653	378(57.9%)	4	275(42.1%)	3	99.2%
Hospital 3	558	323(57.9%)	0	235(42.1%)	1	99.7%
Hospital 4	458	265(57.9%)	0	193(42.1%)	1	99.6%
Hospital 5	185	106(57.3%)	3	79(42.7%)	0	100%
Total	2600	1503	7	1097	9	99.4%

There is a lack of proper supervision and dedicated staff for data cleaning. The absence of a regular supervision schedule for data documentation and cleaning within the hospitals was reported to contribute to incomplete and potentially inaccurate information. Lack of a supervision schedule contributed to poor quality of clinical notes. Data cleaning was reported to be essential in both electronic and manual clinical notes, especially during data entry. Deployment of data officers and proper supervision were reported to improve documentation for prostate cancer.

Lack of proper supervision impairs the quality of clinical notes *“As you know, if nobody is insisting on completeness of clinical notes we continue with our business as usual of writing briefly. I suggest having regular data cleaning sessions and proper supportive supervision to improve the documentation of information obtained regarding patients with prostate cancer and others*” (HCP-5–21)**.** The ongoing transition from manual to electronic clinical notes, often with double data entry, leads to duplication and inconsistencies in the information, highlighting a challenge in the health system’s modernization efforts. The stability and consistency of information in clinical notes were observed despite attending different numbers of patients with prostate cancer across tertiary hospitals. This implies that authorities of hospitals might make proper and timely decisions. It was reported that a few errors do occur in documentation in the two recording systems. Health information management systems (HIMS) were not integrated due to lack of common platform for access of clinical notes between hospitals. All five tertiary hospitals had a duplication of information in clinical notes. This was reported to be caused by the transition from paper-based(manual) to electronic clinical notes, of which some information from clinical notes were entered twice. This exaggerated the magnitude of prostate cancer and misinformed the administration. Efforts to clean institutional databases by removing double entry during migration to electronic records were ongoing however, integration of HIMS across all tertiary hospitals needs policy change. Accurate and consistent information from clinical notes in the integrated HIMS would give clinicians access to the information of patients to avoid repetitions of whatever was done at the referring hospital.

Coexistence of electronic and manual record systems impairs the quality of clinical notes *“We were using paper-based(manual clinical notes) notes so when we shifted to electronic record systems all the all information from clinical notes were entered into the computer, we have noted duplication which happened during data entry, we are trying to clean our database but it will take time and sometimes you find the patients with the same hospital number in the computer are labeled as female for the diagnosis of prostate cancer, and in the clinical notes (manual clinical notes) is labeled as male patient with prostate cancer* ”(HCP-3–11).

### Security information for prostate cancer management

All hospitals had databases for the storage of electronic information from clinical notes and have data storage rooms locked. Whereas two of the hospitals make backups monthly, there are no clear guidelines in the remainder as evinced in Table 4.

**Table 4:** Information on data security for prostate cancer at tertiary hospitals in Tanzania.

Facility	Personnel with access to the data	Who is responsible for data storage?	Presence of database for electronic data storage	Presence of data backups	Frequency of data backups	Guidelines for data storage and backups
Hospital 1	HCP/Managers	Head of the unit	Yes	Yes	Not clear	No guideline
Hospital 2	HCP/Managers	Head of the unit	Yes	Yes	Not clear	No guideline
Hospital 3	HCP/Managers	Head of the unit	Yes	Yes	Monthly	No guideline
Hospital 4	HCP/Managers	Head of the unit	Yes	Yes	Monthly	No guideline
Hospital 5	HCP/Managers	Head of the unit	Yes	Yes	Not clear	No guideline

Tertiary hospitals are using paper-based(manual) cabinets and electronic databases to store patients’ information from clinical notes. It was reported that access to information from clinical notes was limited to the health care providers (HCP) and managers and was found to be password protected. The hospital managers reported that data rooms are locked by key and lock, and the head of the unit is the custodian. Only authorized staff had access to information from clinical notes; this practice builds trust in patients, but it is also a regulatory obligation. All hospital databases for storage of electronic information in clinical notes have storage rooms under key and lock. Whereas two of the hospitals make backups monthly, there are no clear guidelines in the remainder.

Proper storage of clinical notes enhances data security *“We are using both manual and electronic clinical notes s, the clinical notes are stored in medical records, and there is no access for information extraction without approval from the executive director*” (HCP-2–07).

Tertiary hospitals had special rooms with locked cabinets for storage of manual clinical notes. The server for electronic information in clinical notes was highly protected with limited access. Staff was reported to have strong passwords for electronic data protection. There were no data protection officers employed in any of the visited hospitals. Respondents did not know that data protection officers are a profession. It was reported that ICT officers and medical recorders sometimes overlap in data security. Hospital administration was reported to be the controller of data security. A patient who was treated for prostate cancer had signed consent during registration so that their information could be used for research without personal identification. While data security was found to be promising with password protection and locked storage, there were no clear guidelines for data storage backups in some hospitals.

Protection of patients’ information in clinical notes is important for delivery of quality service *“As you know, we are dealing with sensitive information (information about the health of people), We are very strict about providing information from clinical notes as you experienced, you must get approval from hospital administration after ethical review and we must get a copy of the approval to give you clinical notes of our patients*” (HCP-1–01).

## Discussion

This study on data quality for the management of prostate cancer at tertiary hospitals in Tanzania revealed significant findings regarding data completeness and integration, highlighting them as major hurdles to quality-of-care improvement. We found that clinical notes for prostate cancer patients were largely incomplete for most variables. Clinical stage was documented in only 70% of the clinical notes while the Gleason score, crucial for determining the histological pattern of prostate cancer, was reported in only 61.4%. The overall accuracy of information in clinical notes was promising, with a lot of consistency in the documented variables in both electronic and manual clinical notes. These tertiary hospitals have good storage of information in clinical notes and security systems that assure compliance with general data protection regulations [25] however, guidelines for clinical notes storage backups were not clearly stated. Health Information Management System (HIMS) and hospital-based cancer registries were not integrated impairing the continuity of care. Moreover, these HIMS and cancer registries are not integrated across the facilities, limiting continuity of care upon referral. HCPs reported that poor quality of clinical notes was influenced by lack of knowledge, improper supervision, shortage of human resources, coexistence of electronic and manual record systems, data storage and protection of patients’ records. Poor quality of clinical notes has both direct and indirect effects on the effectiveness and efficiency of the healthcare system for the management of prostate cancer [8,30].

The poor documentation of variables like education, marital status, clinical stage, and Gleason score was primarily attributed to the discretion of attending clinicians, as these were often not mandatory on clerkship forms, and a lack of knowledge among some healthcare providers regarding the importance of proper documentation. Healthcare providers, especially medical recorders, sometimes do not document specific disease variables because they do not understand their importance. This was contrary to standard for completeness of clinical notes is recommended that should be above 90% [22,23]. Furthermore, the absence of proper supervision for quality clinical notes meant a lack of feedback on hospital performance, hindering quality improvement efforts [31–33]. Poor data quality in prostate cancer management directly and indirectly impairs treatment outcomes and the overall quality of care for patients [22,34]. Compromised clinical decision-making due to incomplete data can lead to lack of essential diagnostic information which can improve the supply chain for quality service delivery. Timely and accurate decision-making for prostate cancer treatment relies on good quality data, including the patient’s clinical stage and the Gleason score [35].

A significant systemic challenge identified was the lack of integration between Health Information Management Systems (HIMS) and hospital-based cancer registries, both within and across facilities. This fragmentation severely impairs the continuity of care for prostate cancer patients. The lack of integration means that when patients are referred from one hospital to another, there is no seamless data flow, forcing clinicians to retake patient histories and repeat investigations already performed, which causes unnecessary delays in service delivery. These delays can negatively impact patient outcomes, including psychosocial support, which serves as a proxy for quality services. This fragmented approach also compromises the quality of data available for establishing the national pattern and burden of prostate cancer, essential for effective planning and resource allocation [6,36–38]. We also noted poor documentation of whether prostate cancer patients were discussed by multidisciplinary tumor boards, implying that patients might not be benefiting from a teamwork approach in treatment decision-making, which can lead to undertreatment or overtreatment [39].

The coexistence of electronic and manual record systems was found to impair data quality, leading to a substantial duplication of information in 42.1% of clinical notes across tertiary hospitals. This duplication primarily occurred during the transition from manual to electronic systems, which began at different times. Such duplication can exaggerate the reported magnitude of prostate cancer and provide misleading information to administrators [40]. While efforts to clean institutional databases are ongoing, this study highlights the need for well-trained data officers to perform data cleaning, which would reduce duplication and ensure more consistent and reliable information for planning. Implementing structured data capture mechanisms at the point of care could further improve hospital performance [31,41–43] Incomplete information in referral letters, partly due to poor initial documentation, forces receiving hospitals to repeat assessments, causing delays in service delivery and indicating a lack of seamless information flow within the referral system.

Regarding data security, our study observed good practices in tertiary hospitals. All hospitals had databases for electronic data storage and secured data rooms, often locked. Access to patient information was limited to authorized healthcare providers and managers, with password protection for electronic systems and an approval process for information extraction, ensuring compliance with general data protection regulations (GDPR) [44]. However, a key gap was the absence of clear guidelines for data storage backups in several hospitals. Furthermore, no dedicated data protection officers were employed in any of the visited hospitals, with ICT officers and medical recorders sometimes overlapping in data security roles. This study suggests that while security measures are present, a more formalized and structured approach to data governance, including robust backup plans and designated data protection personnel, is necessary as hospitals migrate from manual to electronic records. The study emphasizes the critical need for establishing electronic databases in cancer registries for quality improvement, as information with questionable quality perpetuates a cycle of inadequate information, impairing resource utilization for prostate cancer services [25,44,45].

Overall, the findings of this study underscore that despite the high accuracy of documented information, the low completeness of critical clinical variables, combined with systemic issues such as fragmented HIMS, poor data integration, inadequate supervision, and human resource shortages, presents a considerable struggle to improve healthcare services through data quality improvement in Tanzania [16].The results of this study both align with and add to existing knowledge in the field of health data quality, particularly concerning cancer care in Sub-Saharan Africa (SSA) [8]. Our study corroborates existing knowledge that Health Information Management Systems (HIMS) and hospital-based cancer registries are fragmented and not integrated in Tanzania. The identified factors contributing to poor data quality, such as a lack of knowledge on data quality, improper supervision, shortage of human resources for health (HRH), and heavy workload, align with human, health system, and infrastructure challenges previously reported in SSA [8]. This study reinforces the understanding that poor data quality can mislead results, waste time and resources, impair treatment outcomes, and hinder data use for decision-making. The study aligns with general observations that data from health facilities in Tanzania, like other low and middle-income countries (LMICs), can be questionable in quality, challenging their use for decision-making and other intended benefits [3,8,32,44–46].

### Strength and limitations

The strength of this study lies in its pragmatic mixed-method approach, which integrated both deductive (positivist) and inductive (constructivist) views during thematic analysis. This allowed for a nuanced understanding of data quality concerns by considering both verifiable facts (e.g., security and storage) and healthcare providers’ interpretations (e.g., completeness and accuracy). The methodology systematically assessed data quality concerns at various health system levels and processes, facilitating an understanding of factors crucial for designing appropriate interventions. Data was collected through direct observation from five diverse tertiary hospitals, enhancing the credibility of the results and their transferability to other hospitals in Tanzania facing similar documentation challenges. The purposive selection of participants based on their experience further contributed to the dependability of the findings.

However, this study has limitations, including the potential for social desirability bias and recall bias from interviewed healthcare providers. Its generalizability may be limited to tertiary hospitals and might not fully reflect the challenges faced by primary and secondary level facilities with different resources. Additionally, while key dimensions of data quality were assessed, other aspects such as timeliness and relevance were not explicitly evaluated in the same depth.

## Conclusion

Completeness of clinical notes for prostate cancer care in Tanzanian tertiary hospitals was poor, the accuracy and security of the documented information from clinical notes were promising. The completeness of information in clinical notes is crucial and should be emphasized. Lack of structured forms, shortage of human resources, and fragmented HIMS were contributing factors to poor completeness of clinical notes. Accuracy of information in clinical notes was affected by the duplication of clinical notes, mainly due to the transition from manual to electronic clinical notes. Hospital-based Health Information Management Systems (HIMS) and cancer registries were not integrated, jeopardizing continuity of care. The use of integrated health information system could help to improve data quality in clinical notes and ensure continuity of care across the referral system for cancer care.

## Recommendations

A strong emphasis is needed on capacity building through training and supportive supervision for healthcare providers on the importance and quality of clinical documentation, alongside the deployment of well-trained data officers to perform data cleaning and ensure consistent and reliable information for planning and resource allocation. Furthermore, establishing formalized data governance structures with clear guidelines for data storage backups and considering dedicated data protection officers is essential to enhance data security and integrity, ultimately leading to improved patient outcomes and the optimal utilization of resources in prostate cancer services.

## Supporting information

S1 Text Consolidated criteria for reporting qualitative research (COREQ) checklist … 4. (DOCX)
