# Supplementary material for: Challenges of data quality for the management of prostate cancer in Tanzania: A hurdle towards improving the quality of service
Source: PLOS Glob Public Health. 2025 Dec 4;5(12):e0005579. doi: 10.1371/journal.pgph.0005579 (PMC12677768; doi:10.1371/journal.pgph.0005579)
Supplement: S1 Text — (DOCX) [file pgph.0005579.s001.docx]

S1_Text.

A checklist of items that should be included in reports of qualitative research. You must report the page number in your manuscript where you consider each of the items listed in this checklist. If you have not included this information, either revise your manuscript accordingly before submitting or note N/A.

**Title:** **Challenges of data quality for the management of prostate cancer in Tanzania: A hurdle towards improving the quality of service**

| Topic | Item No. | Guide Questions/Description | | Reported on  Line No. |
| --- | --- | --- | --- | --- |
| **Domain 1: Research team and reflexivity** | |  | | |
| Personal characteristics | | | | |
| Interviewer/facilitator | | 1 | Which author/s conducted the interview or focus group?  Obadia Nyongole, Nathanael Sirili, Gasto Frumence, David Urassa,Bruno Sunguya | Authors’ contributions |
| Credentials | | 2 | What were the researcher’s credentials? E.g. PhD, MD, Masters in health sciences | Method, paragraph of qualitative data collection |
| Occupation | | 3 | What was their occupation at the time of the study?  All were academic staff at MUHAS | Method, paragraph of qualitative data collection |
| Gender | | 4 | Was the researcher male or female? Males | Cover page |
| Experience and training | | 5 | What experience or training did the researcher have? On average of six years | Method, paragraph of qualitative data collection |

Relationship with participants

| Relationship established | 6 | Was a relationship established prior to study commencement? | Human Participants Research Checklist, Informed consent |
| --- | --- | --- | --- |
| Participant knowledge of the interviewer | 7 | What did the participants know about the researcher? e.g. personal goals, reasons for doing the research | Human Participants Research Checklist, Informed consent |
| Interviewer characteristics | 8 | What characteristics were reported about the inter viewer/facilitator? e.g. Bias, assumptions, reasons and interests in the research topic | Method, paragraph of qualitative data collection |

**Domain 2: Study design**

Theoretical framework

| Methodological orientation and Theory | 9 | What methodological orientation was stated to underpin the study? Thematic analysis was conducted | Data analysis, a paragraph for qualitative analysis |
| --- | --- | --- | --- |

Participant selection

| Sampling | 10 | How were participants selected? Purposive sampling was used | Study population |
| --- | --- | --- | --- |
| Method of approach | 11 | How were participants approached? Face-to-face | Qualitative data collection |
| Sample size | 12 | How many participants were in the study? 25 IDIs | Study population |
| Non-participation | 13 | How many people refused to participate or dropped out? Reasons? None | Ethics statement and study population |

Setting

| Setting of data collection | 14 | Where was the data collected? Data were collected at hospital settings | Study setting |
| --- | --- | --- | --- |
| Presence of non-participants | 15 | Was anyone else present besides the participants and researchers? No | Ethics statement |
| Description of sample | 16 | What are the important characteristics of the sample? e.g. demographic data, date | Results, Sociodemographic information of qualitative interview participants |

Data collection

| Interview guide | 17 | Were questions, prompts, guides provided by the authors? Was it pilot tested? YES | Quantitative data collection and Qualitative data collection |
| --- | --- | --- | --- |
| Repeat interviews | 18 | Were repeat inter views carried out? If yes, how many? NO | Qualitative data collection |
| Audio/visual recording | 19 | Did the research use audio or visual recording to collect the data? YES | Qualitative data collection |
| Field notes | 20 | Were field notes made during and/or after the interview or focus group? YES | Qualitative data collection |
| Duration | 21 | What was the duration of the inter views or focus group? In-depth interviews (IDIs) lasted 15–30 minutes | Qualitative data collection |
| Data saturation | 22 | Was data saturation discussed? YES, Saturation of information was reached from 23^rd^ to 25^th^ participants. | Qualitative data collection |
| Transcripts returned | 23 | Were transcripts returned to participants for comment and/or correction? No | Qualitative data collection |

**Domain 3: analysis and findings**

Data analysis

| Number of data coders | 24 | How many data coders coded the data? Three coders | Data analysis, a paragraph for qualitative analysis |
| --- | --- | --- | --- |
| Description of the coding tree | 25 | Did authors provide a description of the coding tree? Yes | Data analysis, a paragraph for qualitative analysis |
| Derivation of themes | 26 | Were themes identified in advance or derived from the data? Derived from data | Data analysis, a paragraph for qualitative analysis, |
| Software | 27 | What software, if applicable, was used to manage the data? Yes. NVivo 14 software was used | Data analysis, a paragraph for qualitative analysis, |
| Participant checking | 28 | Did participants provide feedback on the findings? | N/A |

Reporting

| Quotations presented | 29 | Were participant quotations presented to illustrate the themes/findings? Was each quotation identified? YES e.g. participant number | Results from third paragraph |
| --- | --- | --- | --- |
| Data and findings consistent | 30 | Was there consistency between the data presented and the findings? YES | Results from second paragraph |
| Clarity of major themes | 31 | Were major themes clearly presented in the findings? YES | Results from second paragraph |
| Clarity of minor themes | 32 | Is there a description of diverse cases or discussion of minor themes? YES | Discussion |

Developed from: Tong A, Sainsbury P, Craig J. Consolidated criteria for reporting qualitative research (COREQ): a 32-item checklist for interviews and focus groups. International Journal for Quality in Health Care. 2007. Volume 19, Number 6: pp. 349 – 357
